# Adaptable Phosphate Networks towards Robust, Reprocessable, Weldable, and Alertable-Yet-Extinguishable Epoxy Vitrimer

**DOI:** 10.34133/2022/9846940

**Published:** 2022-10-06

**Authors:** Jia-Hui Lu, Zhen Li, Jia-Hui Chen, Shu-Liang Li, Jie-Hao He, Song Gu, Bo-Wen Liu, Li Chen, Yu-Zhong Wang

**Affiliations:** School of Chemical Engineering, The Collaborative Innovation Center for Eco-Friendly and Fire-Safety Polymeric Materials (MoE), National Engineering Laboratory of Eco-Friendly Polymeric Materials (Sichuan), State Key Laboratory of Polymer Materials Engineering, Sichuan University, Chengdu 610064, China

## Abstract

Covalent adaptable networks (CANs) combine the uniqueness of thermoplastics and thermosets to allow for reprocessability while being covalently crosslinked. However, it is highly desirable but rarely achieved for CANs to simultaneously demonstrate reversibility and mechanical robustness. Herein, we report a feasible strategy to develop a novel epoxy vitrimer (EV) composed of adaptable phosphate networks (APNs), by which the EVs exhibit promising mechanical properties (tensile strength of 62.5 ~ 87.8 MPa and tensile modulus of 1360.1 ~ 2975.3 MPa) under ambient conditions. At elevated temperatures, the topology rearrangement occurs relied on phosphate transesterification, which contributes to the shape memory performance, self-healing, reprocessing, and welding behaviors. Moreover, the incorporation of APNs allows for improvements in anti-ignition and also the inhibition of both heat release and smoke generation to avoid empyrosis, asphyxiation, and toxication during burning, showing expected intrinsic fire safety. Thermal, mechanical properties, and flame retardancy of the reprocessed EVs after hot pressing are very close to those of the original EVs, which is attributed to the sufficient reversibility of APNs. Accordingly, combining the aforementioned features, EVs are manufactured as flame-triggered switches for fire alarms, which symbolizes the innovative development of high-performance covalent adaptable polymeric materials.

## 1. Introduction

Stimuli-responsive polymeric materials have attracted substantial attention due to the surging development of intelligent devices such as smart actuators, soft robotics, tissue scaffolds, and unfolder devices [1–4]. Thermosetting stimuli-responsive materials are preferable for their mechanical properties and dimensional stabilities [5] compared to thermoplastic ones. However, there is an inevitable difficulty for thermosets in further reprocessing after curing, which is ascribed to their permanent crosslinked networks [6]. To date, the development of vitrimers [7], or associative CANs, with glass-like fluidity [8], which feature dynamically exchangeable bonds and allow rearrangement of the networks, has given a solution to this issue. From this vantage point, a bond-exchange reaction triggered by heat enabled thermosetting materials with shape memory behavior, self-healing, reprocessability, and weldability while maintaining the network integrity [9–11]. Thus, a growing body of research has focused on various types of dynamic covalent mechanisms, such as transalkylation [12], transamination [13], olefin metathesis [9], boronic ester transesterification [14], disulfide exchange [15], imine exchange [16], urea exchange [17], and siloxane-silanol exchange reactions [18].

Nonetheless, compared to conventional thermosets with permanent crosslinks, vitrimers are prone to exhibiting intrinsic fragility or limited stability due to their reversible nature, which may impair their service performance, including durability, particularly when subjected to multiple deformations or accidental damages. Previous research has focused on adjusting the crosslinking density [19] or enhancing network stiffness by introducing conjugated aromatic segments [20]. These approaches, however, always result in a reduction in bond-exchange reactivities, bringing about a conflict between inherent robustness and rapid response. Introducing sacrificial bonds [21, 22] to vitrimers is another well-accepted choice to enhance the mechanical properties by dissipating external energy. However, the approaches are restricted to complicate molecular designs. Moreover, the utilization of bond-exchange catalysts further reduces thermal stabilities [23] and raises the possibility of toxicity or incompatibility concerns [24]. Thus, Van Lijsebetten et al. [25] proposed an internal catalysis strategy for attaining intrinsic robustness and fast exchange through a neighboring-group-participation mechanism. Hao et al. [26] used *β*-hydroxyester with a hydroxyl group neighboring the ester linkage to create EV with high *T*_*g*_ (135°C), exceptional tensile strength (94 MPa), and high reversible rate (10 min at 190°C). Adjaoud et al. [27] created entirely biobased benzoxazine vitrimers with relatively high *T*_*g*_ ranging from 143 to 193°C and appropriate dynamic characteristics, which were ascribed to internally catalyzed transesterification processes. Consequently, these internal catalyzed systems attracted special attention to create highly reversible polymeric networks with superior mechanical properties and thermal stabilities.

To date, the dynamic features belonging to the phosphate bond have been reported through internal transesterification in ribonucleotides [28] and hydrogels [29]. Compared to conventional carboxylate transesterifications, there exist three stable but divergent bonds in addition to the P=O double bonds in phosphate [30], which enables adaptable phosphate networks (APNs) with versatile structural designability, from main- to side-chain modification. From this point of view, adaptable phosphates serve as the ideal candidate for fabricating robust but highly reversible CANs. Herein, Majumdar et al. [31] initially presented a novel APN that was chemically bonded between phosphate triester and polytetrahydrofuran, which endowed the thermoset with catalyst-free reprocessability.

Moreover, concerning the high flammability, together with the massive release of heat and smoke once ignited, polymeric materials with flame retardancy are in urgent need in real applications [32]. Benefited from high flame-retardant efficiency in both gaseous and condensed phases, versatile phosphorus-containing flame retardants have been well-established, which molecular structures range from small molecule to polymeric ones, and the incorporation methods include physical incorporation (additives) and chemical introduction ((co)polymerizing, curing, grafting, etc.) [33–36]. Although diverse additive flame retardants have been successfully employed in different polymeric materials, state-of-the-art applications need intrinsically fire-safety materials [37] that are durable and possess comprehensive properties. Phosphates (or phosphate esters), as typical phosphorus-containing groups showing acceptable thermal stability, designability, and reliability, have been integrated into the molecular chain of polymers to endow their expected flame retardancy [38]. For example, Feng and Li [39] reported a novel phosphate-diester-based vitrimer with intrinsic flame retardancy and good mechanical properties. Liu et al. [40] studied a novel thermoset containing APNs from crosslinking of the bio-derived itaconic-based epoxy monomer and fully biobased phytic acid, which showed recyclability and flame retardancy. These studies addressed ways to improve the recyclability and flame retardancy of polymeric materials; however, vitrimers with comprehensive promising properties, particularly a delicate balance between thermal stability, mechanical properties, and reprocessability, are in great demand.

In this work, a novel catalysis-free EV composed of APNs was designed and fabricated by introducing a phosphate-based ionic liquid named 1-butyl-3-methylimidazolium diphenyl phosphate ([Bmim]DPPOO) as a curing agent for diglycidyl ether of bisphenol A (DGEBA), which exhibited a combination of mechanical robustness, topological reversibility, and flame retardancy. The curing process was verified between phosphate groups and DGEBA through anionic ring-opening polymerization to obtain crosslinked networks with reversible *β*-hydroxy phosphate ester bonds. Through phosphate transesterification, the EVs demonstrated superior shape memory performance, self-healing capability, reprocessability, and weldability. Furthermore, EVs were included in the smart fire-alarm device due to their high thermal stability, flame tolerance, and rapid recovery.

## 2. Results and Discussion

### 2.1. Curing, Transesterification, and Mechanical Properties

**Figure 1:**
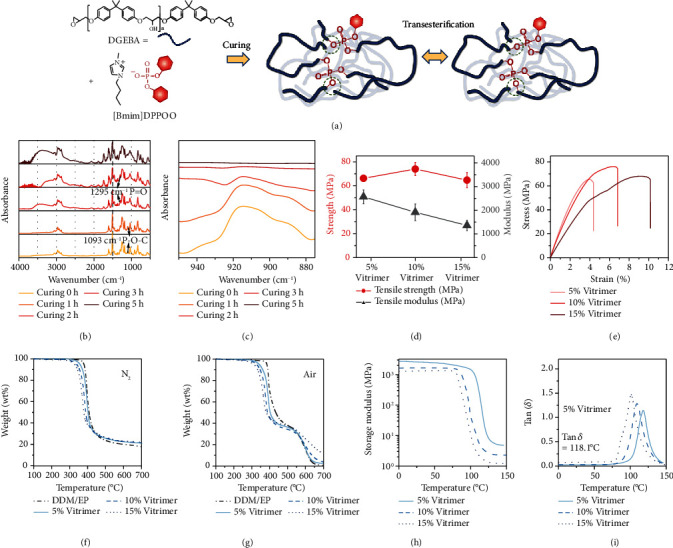
Curing process and the thermally reversible phosphate transesterification of EVs (a). Time-dependent FT-IR spectra of 15% vitrimer at 180°C (b). The partially enlarged detail at approximately 940~880 cm^−1^ of the time-dependent FT-IR spectra of 15% vitrimer at 180°C (c). Tensile strength and modulus of the vitrimers (d). Tensile stress-strain curves (e). TGA curves for EP cured by DDM and EVs in N_2_ and air atmospheres (f, g). Storage module in DMA testing as a function of temperature (h). Tan *δ* curves of the EVs as functions of temperature (i).

At ambient temperature, the phosphorus-containing ionic liquid [Bmim]DPPOO remained in a liquid state with low viscosity, and its chemical structure was confirmed by FT-IR and NMR spectra (Figure S1). To our knowledge, ionic liquids have been utilized as catalytic curing agents in fabricating high-performance epoxy resin [41, 42]. Here, the curing behavior of [Bmim]DPPOO and DGEBA was studied by nonisothermal differential scanning calorimetry (DSC), and the gelation process was investigated through a dynamic rheometer, as depicted in Figure S2. The exothermic peaks were observed at approximately 180°C, while the curing activation energies (*E*_*a*_) of 5% vitrimer, 10% vitrimer, and 15% vitrimer were 124.0, 103.8, and 90.4 kJ·mol^−1^, respectively. The relatively high curing temperatures and high *E*_*a*_*s* were attributed to the electrical withdrawal effect and the large steric hindrance of the phenoxy-substituted phosphate groups. Further, the curing process of 15% vitrimer was illustrated by time-dependent FT-IR at 180°C. As shown in Figures 1(b) and 1(c), the characteristic absorption peaks of P=O (1295 cm^−1^) and P-O-C (1093 cm^−1^) were maintained, indicating the existence of phosphate ester groups. The EVs were completely cured because of the disappearance of the epoxide band (912 cm^−1^) and the generation of hydroxyl groups (3350 cm^−1^) as well as carbonyl groups (1735 cm^−1^) [43]. To illustrate the chemical structures of the EVs and further clarify the curing mechanism, XPS was conducted, and the high-resolution spectra were interpreted as shown in Figure S3. Nitrogen derived from the N-(C)_3_ structure was observed at rather low binding energies (399.6 eV) due to the electron-donating effect of the butyl substituents. Importantly, the positive nitrogen atoms C-N=C (401.5 eV) belonging to the imidazolium structures indicated that the imidazole groups initiated the curing reaction through the ring-opening mechanism. The signal at 133.6 eV for P 2p was assigned to the predominant P-O-C structures that were covalently bonded to the DGEBA chain. Consequently, the EVs with APNs were fabricated through anion polymerization initiated by [Bmim]DPPOO; the curing process and the primary structures of the cured EVs were illustrated in Scheme S1. The dynamic phosphate bonds embedded in the EV networks allowed phosphate transesterification, which resulted in thermal-induced network topological rearrangement.

As seen in Figure S4(a), the vitrimers with varying contents of the curing agents exhibited transparent and uniform appearance without phase separation, indicating the generation of homogeneous EV networks. However, when the ratio of [Bmim]DPPOO was increased, the color changed from pale yellow to dark brown, which was ascribed to the chromogenic impact of the imidazole groups. The crosslinked networks were verified by swelling tests, which showed volumetric swelling without dissolving, as demonstrated in Figure S4(b). The corresponding swelling ratios of 15% vitrimer in common solvents were illustrated in Table S1. Additionally, gel content measurement at elevated temperature was tested within 48 hours by solvent extraction with acetone, in which all the starting materials were soluble. In Figure S4(c), the gel contents of 5% vitrimer, 10% vitrimer, and 15% vitrimer were 99.1%, 98.5%, and 98.1%, respectively, indicating the formation of well-cured crosslinked networks. The tensile properties of EVs were shown in Figure 1(d). In this case, the tensile modulus of 5% vitrimer was 2569.5 MPa, with a tensile strength of 66.2 MPa, verifying that the stiff crosslinked networks with strong mechanical properties. However, 15% vitrimer was flexible with rather large elongations at breaks (in Figure 1(e)) and decreased tensile modulus (1360.1 MPa), which was attributed to the increased amount of the flexible phosphate segments. Among these specimens, 10% vitrimer showed the best tensile properties with a tensile modulus of 1916.9 MPa and tensile strength of 74.0 MPa, owing to the appropriate amounts of phosphate groups and adequate crosslinking densities.

Thermogravimetric analysis (TGA) was conducted to explore the thermal decomposition behaviors of EVs. The curves are shown in Figure 1(f) (N_2_) and Figure 1(g) (air), while the corresponding data were collected in Table S2. Here, the temperature of 5% weight loss (*T*_5%_) is defined as the initial decomposition temperature, whereas the temperature at which the maximum weight loss occurs is referred to as *T*_max_. Under the N_2_ atmosphere, the *T*_5%_ values of all the EVs were lower than that of the epoxy resin cured with DDM, owing to the initial decomposition of organic phosphate [44] as well as the alkyl side chains with poor thermal stability. The value of *T*_5%_ decreased when the proportion of [Bmim]DPPOO was increased. Nonetheless, the *T*_5%_ of all examined samples exceeded 320°C, even in a thermal-oxidative environment, showing satisfactory thermal stabilities. The lower *T*_max_ of the vitrimers was ascribed to the enhanced decomposition and dehydration effects afforded by the phosphate groups. Notably, 15% vitrimer produced a residue of 22.2 wt%, which was much higher than that of the DDM/EP reference (17.1%), indicating that the phosphate group facilitated the carbonization process. As an illustration in Table S2, when the content of [Bmim]DPPOO increased, the increased weight of residues was observed.

Thermal-mechanical behaviors of the EVs were evaluated by dynamic mechanical analysis (DMA). Specifically, the storage modulus (*E*′) and loss factor (tan *δ*) as functions of temperature are illustrated in Figures 1(h) and 1(i), while the data were summarized in Table S3. In the glassy state, 5% vitrimer showed the highest *E*′ among all the vitrimers, with a value of 2528.3 MPa, revealing highly rigid networks. For 10% vitrimer and 15% vitrimer, the values of *E*′ in the glassy state were 1646.1 MPa and 1226.7 MPa, respectively, which was consistent with the results obtained from the tensile tests since the integration of phosphate contributes to flexible crosslinked networks. Additionally, *α* relaxation transition temperature (*T*_*α*_), which is defined as the peak temperature of the dissipation factor, reveals thermal transition behaviors of EVs from glassy to a rubbery state. *T*_*α*_ occurred at 118.1°C for 5% vitrimer, 109.8°C for 10% vitrimer, and 101.2°C for 15% vitrimer. The decreased *T*_*α*_ was attributed to the enhanced segmental movement and the plasticizing effect enabled by the flexible phosphate ionic liquid [45]. Meanwhile, the crosslinking density (*υ*_*e*_) was calculated using the modulus of the “rubbery plateau,” which corresponded to the constant *E*′ beyond *T*_*α*_. When the content of [Bmim]DPPOO increased, the crosslinking density of EVs was reduced. The phenomenon was explained that increasing the contents of the curing agent promoted the initial reactions and yielded ring-opening products in the curing process, in which the epoxy groups were consumed to fabricating adaptable phosphate segments rather than crosslinking. Accordingly, the APNs were customized from stiff to flexible, and the viscoelasticity of the EVs was feasibly regulated by changing the proportions of [Bmim]DPPOO.

### 2.2. Flame Retardancy

**Figure 2:**
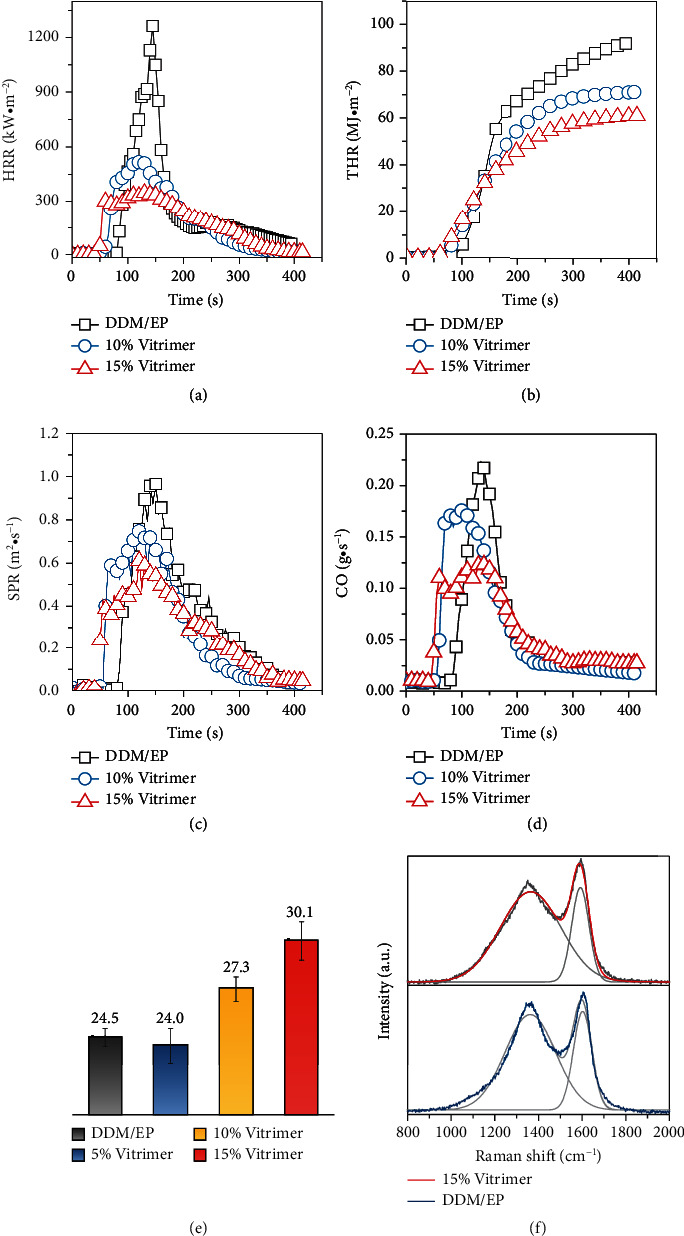
Flame retardancy of DDM/EP and EVs: HRR curves (a); THR curves (b); SPR curves (c); CO production (d); LOI values (e); Raman spectroscopy of burning residues in cone calorimetry test (f).

Cone calorimetry was used to determine the flame retardancy of the EVs [46], in which heat release, smoke production, and CO generation were reviewed. As illustrated in Figures 2(a) and 2(b), the peak heat release rate (pHRR) and total heat release (THR) were significantly reduced with the incorporation of [Bmim]DPPOO. Notably, as shown in Table S4, the pHRR decreased by 74.8% (for 15% vitrimer) and 63.6% (for 10% vitrimer) compared to the epoxy resin cured by DDM. Smoke, which is produced in the burning of polymeric materials, often causes more catastrophic injuries and fatalities in fire disasters than heat [47]. The smoke production rate (SPR) included in Figure 2(c) revealed that the peaks of SPR were 0.75 m^2^·s^−1^ (10% vitrimer) and 0.55 m^2^·s^−1^ (15% vitrimer), referring to remarkable reductions in comparison to those of DDM/EP. Simultaneously, the CO production was significantly suppressed in EVs, as shown in Figure 2(d). Consequently, cured with [Bmim]DPPOO, the EVs demonstrated exceptional flame retardancy and smoke inhibition in burning tests. As shown in Figure 2(e), the limiting oxygen index (LOI) of the EVs improved as the [Bmim]DPPOO content increased; nevertheless, the 5% vitrimer demonstrated poor flame retardancy due to the insufficient gas-phased activity provided by phosphate groups [48]. Furthermore, burning residues after cone calorimetry were studied using Raman spectroscopy to elucidate the condensed flame-retardant mechanism. In Figure 2(f), the ratio of *I*_*D*_/*I*_*G*_ (see the Supplementary Materials) of 15% vitrimer was much greater than that of DDM/EP, which meant the formation of smaller carbonaceous microstructures [49] with an effective thermal shielding effect and higher fire safety features. Thus, APNs enhanced the flame retardancy of EVs.

### 2.3. Reprocessing

**Figure 3:**
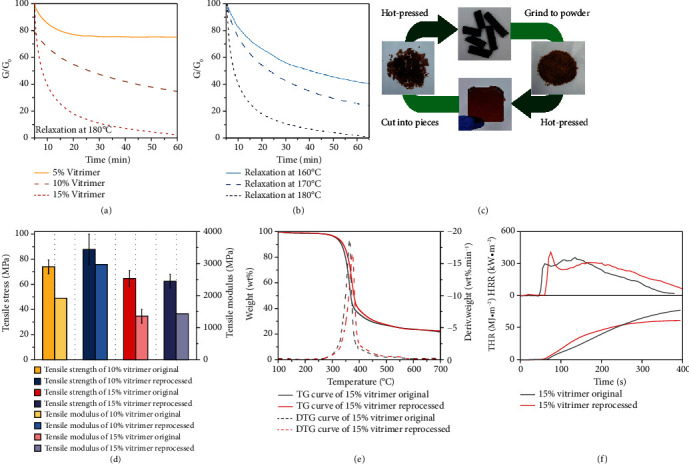
The stress-relaxation behaviors of EVs with different proportions of [Bmim]DPPOO (a). Stress relaxation curves of 15% vitrimer at varying temperatures (b). Recycling scheme of 15% vitrimer (c). The reprocessing properties of vitrimers: tensile results (d) and TGA results under N_2_ atmosphere (e). Cone calorimetric results of the reprocessed 15% vitrimer (f).

Generally, traditional thermosets are inherently difficult to reprocess owing to their three-dimensional crosslinked networks via permanent covalent bonds. By introducing APNs, the EVs rearranged the topology upon phosphate transesterification at the elevated temperature, which further permitted recyclability for the cured EVs. Stress relaxation tests provide insights into viscoelastic behaviors and reveal the reversible features of polymeric materials. As illustrated in Figure 3(a), the stress relaxation behavior of the EVs with different contents of [Bmim]DPPOO was studied in tensile mode. At 180°C, the relaxation was promoted by increasing the phosphate content. For 15% vitrimer, the residual normalized relaxation modulus (*G*/*G*_0_) was only 1.5%, showing substantial network rearrangements and with excellent reprocessability. Generally, the increase of crosslinking densities and the decrease of APNs both have negative influences on stress relaxation, because of the restricted topological rearrangements. Thereby, 10% and 5% vitrimers revealed gentle relaxation rates, with *G*/*G*_0_ values of 34.4% and 74.9% after 60 min, respectively. Figure 3(b) showed the temperature-determined stress relaxation behaviors of 15% vitrimer, which demonstrated that the relaxation rate was accelerated at high temperatures, owing to the increased segmental motions. To further elucidate the stress relaxation behavior and reprocessability for the EVs, relaxation time (*τ*^∗^) [50], when the time for *G*/*G*_0_ = 1/*e*, was recorded, while the bond exchange activation energy (*E*_*a*_) and the topology freezing transition temperature (*T*_*v*_) [51] of 15% vitrimer were calculated via the Arrhenius equation and Maxwell equation, as shown in Figure S5 and Table S5. Consequently, the *E*_*a*_ value was 86.4 kJ·mol^−1^, and the *T*_*v*_ value was 91.4°C for 15% vitrimer. As depicted in Figure 3(c), the 15% vitrimer, which was cut into random fragments or pulverized, was able to be reprocessed through hot pressing at 190°C for 0.5 h. This condition was chosen to optimize the final qualities while minimizing thermal degradation. Additionally, since the phosphate transesterification occurred relied on the internal catalysis chemistry that the phosphate moieties were only exchanged but not consumed during reprocessing, multiple reprocessing of 15% vitrimer was achieved. As manifested in Figure S6, a transparent appearance without macroscopical defects was observed. However, 5% vitrimer failed to reprocess due to a lack of reversible phosphate linkages (as shown in Figure S7).

Although dynamic covalent bonds impart recyclability to the EVs, property impairment is always a problem for the recycled specimens, imputed to the decreased crosslinking densities, inevitable thermal degradation, and inadequate reversibility. Accordingly, in this section, tensile tests (for 10% and 15% vitrimers), thermogravimetric analysis (for 10% and 15% vitrimers), and cone calorimetry (for 15% vitrimer) were implemented to determine the recyclable efficiency. In Figure 3(d) and Table S6, for the reprocessed 10% vitrimer, the tensile strength recovered to 87.8 MPa, while the tensile modulus was 2975.3 MPa, exceeding the original tensile results, which was due to further crosslinking and postcuring reactions during the reprocessing procedure. Additionally, the decreased elongation values at break also verified the postcuring reactions. The tensile strength and modulus of the 15% recycled vitrimer were 62.5 MPa and 1433.6 MPa, respectively, which was comparable to that of the original samples (64.7 MPa and 1360.1 MPa). Notably, the TGA results of recycled 15% vitrimer illustrated that there was no deterioration of the thermal decomposition behavior, since the TGA and DTG curves almost overlapped for the original and reprocessed sample, as shown in Figure 3(e). Further, the thermal decomposition behavior of the recycled 10% vitrimer was comparable to that of the original samples, as shown in Figure S8. These experiments indicated that the reprocessing procedure had negligible effects on the mechanical and thermal properties. Herein, the excellent recyclability of the EVs was attributed to the reasonable crosslinking densities, preferred thermal (oxidative) stabilities, and highly reversible topological rearrangements. Moreover, for the EVs, the viscosity changed following the Arrhenius law and maintaining the network integrity, rather than undergoing the abrupt viscosity drop, avoiding the possible decreases in molecular weight during reprocessing. Additionally, phosphate groups were chemically bonded into the molecular chain, imparting the vitrimers with inherent and long-lasting flame retardancy. Thus, the HRR and THR values of the recycled 15% vitrimer were respected to those of the original sample, as shown in Figure 3(f).

### 2.4. Phosphate Transesterification Mechanism

**Figure 4:**
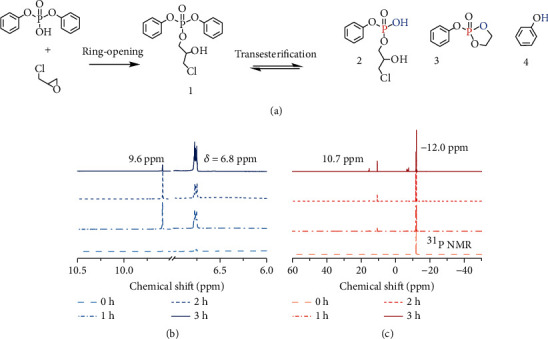
Phosphate transesterification mechanism. Ring-opening and the phosphate transesterification reactions between diphenyl hydrogen phosphate and epichlorohydrin as the model compounds (a). NMR spectroscopy of model compounds at different treatment times at 90°C for ^1^H (b) and ^31^P (c).

Model compounds, including monofunctional epoxy epichlorohydrin (ECH) and diphenyl hydrogen phosphate, were utilized to clarify the mechanism of phosphate transesterification since cured multifunctional EVs cannot be easily characterized. After stoichiometric mixing of the model compounds, ring-opening reactions occurred between ECH and diphenyl hydrogen phosphate through electrophilic addition, generating *β*-hydroxy phosphate, which was confirmed by liquid chromatography-mass analysis in Figure S10 in the Supplementary Materials, and the structure is shown in Figure 4(a) structure 1. Upon heating, phosphate transesterification proceeded (1 ~ 4 h) through *β*-hydroxy participated reaction, which yielded new phosphates with different substituents as well as the new hydroxy products. The dynamic nature of the phosphate was verified through the ^1^H NMR spectrum, where phenyl phosphate ester (structure 2), alkyl phosphate ester (structure 3), and phenol (structure 4) could be observed. The proton signals appeared at 6.8 ppm and 9.6 ppm corresponding to the newly formed alkyl phosphate and phenolic hydroxyl, as shown in Figure 4(b). Additionally, in Figure 4(c), the signal of ^31^P NMR that appeared at approximately 10.7 ppm was attributed to the phosphate ester with a new chemical environment. Chemical structures of *β*-hydroxy phosphate and the transesterification products were clearly proven by MS analysis, as recorded in Figure S10. Thus, the phosphate transesterification was verified by the model compounds.

### 2.5. Shape Memory, Self-Healing, and Welding Performance

**Figure 5:**
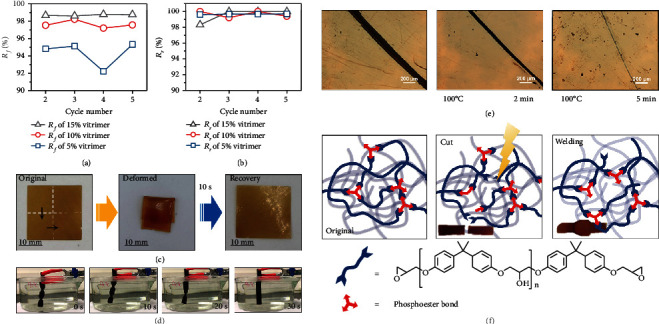
The *R*_*f*_ (a) and *R*_*r*_ (b) result from the shape memory cycles of the vitrimers. Digital photos of the shape recovery behavior of the origami-like 5% vitrimer (c). The shape memory behavior of the twist-like 15% vitrimer under hot water (90°C) (d). Self-healing process for the scratched 15% vitrimer at 100°C (e). Illustration of the self-healing process via phosphate transesterifications, and embedded photos denote the welding-healing process for the broken 15% vitrimer (f).

In general, topological rearrangement occurs when the temperature reaches *T*_*g*_ and becomes much more pronounced when the temperature exceeds *T*_*v*_ [52]. The *T*_*g*_*s* of the vitrimers ranged from 74.6°C to 85.2°C, as illustrated in Figure S9, while the *T*_*v*_ of these phosphate-based vitrimers was predicted to be 91.4°C. Thus, to evaluate the shape memory properties, the fixing temperature was set to 30°C, and the recovery temperatures for 15%, 10%, and 5% vitrimers were 90°C, 100°C, and 120°C, respectively. The thermally induced shape memory cycle of EVs is illustrated in Figures 5(a) and 5(b). The fixing ratio (*R*_*f*_) of vitrimers increased when the fraction of [Bmim]DPPOO was raised since the reversible bond enhanced the elastic energy conservation to fix the temporary conformation. The recovery ratio (*R*_*r*_), on the other hand, is dependent on permanent crosslinked moieties. In this regard, the associative phosphate bonds and the crosslinked networks allowed the molecules to completely release their internal stress, enabling all *R*_*r*_ values to approach 100%. As mentioned above, the phosphate-based EVs revealed excellent shape memory effects with high *R*_*f*_ (92.2~98.8%) and *R*_*r*_ values (98.3~100%). Moreover, to illustrate thermally induced shape recovery behavior, a 5% vitrimer film was folded and then placed in a 100°C heating panel. As shown in Figure 5(c), following thermal equilibration, the folded sample reverted to its original shape within 10 s. Furthermore, not only did the reversible shape-memory transitions occur in the film specimens but were also observed in the bulk samples, with a complete recovery from a 1080° twisting to the original shape in hot water (90°C), indicating that the EVs were suitable for underwater working conditions, as illustrated in Figure 5(d).

Brittle polymeric materials (such as epoxy resin) were prone to scratching or developing macroscopic fractures, leading to short service life. Self-healing and welding endow efficient repairment which enhances materials with longevity and stability [53, 54]. With externally applied pressure, thermally induced topological rearrangement enabled fast welding between the fracture surfaces. In this work, self-healing was evaluated on a blade-cut film specimen for 15% vitrimer at 100°C, and the healing process is documented in Figure 5(e). The breadth of scratch dropped rapidly and vanished within 5 min, demonstrating an extremely high capacity for self-healing. Furthermore, welding experiments were conducted by applying pressure to fractured samples at 190°C/0.5 h. As we can see in Figure 5(f), the welding efficiency was superior since 15% vitrimer was fused without discernible boundaries, relying on the rapid phosphate transesterification reactions.

### 2.6. Flame-Triggered Fire Alarm

**Figure 6:**
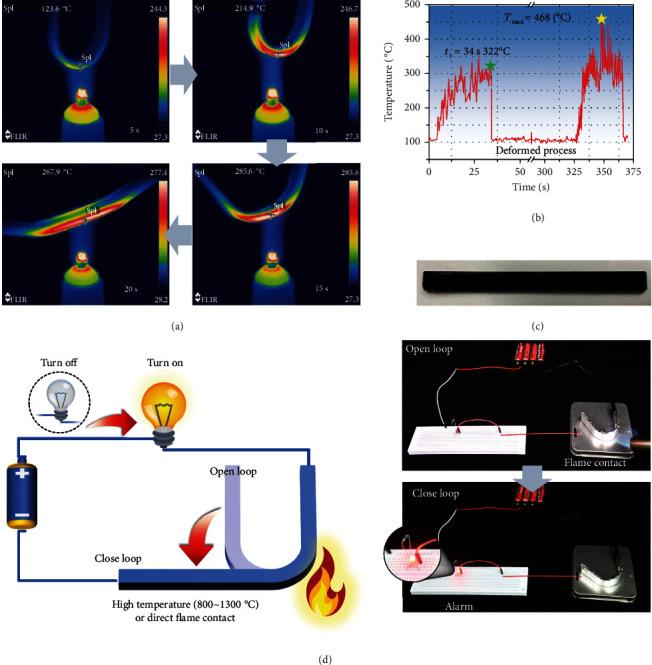
Infrared imaging of the shape and temperature evolution triggered by an alcohol lamp during the recovery process of 15% vitrimer (a). The maximum temperature of vitrimer during the deformation and recovery process (b). Optical images of the flame-triggered shape memory vitrimer after three cycles (c). The flame-triggered alarm: schematic diagram and digital photos for its working process (d).

To the best of our knowledge, conventional thermally induced shape memory polymers fail to perform beyond their specified maximum operating temperatures, such as *T*_*g*_, heat deflection temperature (HDT), or *T*_*m*_. However, the designed EVs illustrated high-temperature response behavior, even when triggered by flame, which benefited from their excellent thermal stability (flame retardancy) and rapid recovery. Shape memory performance was studied by using an alcohol light and captured by an infrared camera, as shown in Figure 6(a). The evolution of the maximum temperature throughout the deformation and recovery process was depicted in Figure 6(b). The results showed that the peak value reached 468°C, implying the excellent fire safety endowed by the APNs. Additionally, following three cycles of flame-triggered shape memory, the resultant vitrimers retained their entire morphology without burn down or even fracture by fire, illustrated in Figure 6(c), versatile high-temperature adaptability, exceptional flame retardancy, and extremely fast deformation recovery. Subsequently, a fire alarm device was fabricated as depicted in Figure 6(d) and Video S1. Specifically, the 15% vitrimer specimen wrapped in aluminum foil was assembled as a switch in an electrical circuit, which featured a DC power supply, a 100 *Ω* resistor, a luminous diode (LED), and cables. Before applying the ignitor, the prebent vitrimer was in a static condition due to the alerting circuit being disconnected. However, after exposure to the flame, the specimen quickly reverted to its previous shape and activated the warning circuit, as proven by the illumination of the LED. Additionally, there are various advances and benefits associated with the use of this fire alarm equipment. First, the materials used for fire alarms are readily recyclable due to the dynamic character of the vitrimer. Second, the improved mechanical robustness and thermal stability aided in the performance and stability of the flame-triggered system. Third, the vitrimer was triggered only at quite high temperatures (>90°C) or under flame conditions, avoiding false alarms caused by temperature or smoke, as conventional temperature/smoke alarms were performed.

## 3. Conclusions

To date, it is highly desirable but rarely achievable for vitrimers to simultaneously perform fast reversibility and mechanical robustness, much less to construct a multifunctional actuator with intrinsic fire safety. Herein, we designed and prepared a novel EV by introducing APNs via anion polymerization, which was implemented between the commercially available DGEBA and phosphorus-containing ionic liquid termed [Bmim]DPPOO. Such EVs exhibited promising mechanical properties (tensile strength and modulus high as 87.8 MPa and 2975.3 MPa, respectively), which were comparable to those of conventional DGEBA/DDM curing systems. The internal *β*-hydroxy participated phosphate transesterification allowed the EVs to realize thermal-induced topological rearrangement towards shape memory performance, self-healing, reprocessing, and welding capacities. Furthermore, the phosphate moieties of APNs allowed for improvements in anti-ignition, yet the inhibition of both heat release and smoke generation to avoid empyrosis, asphyxiation, and toxication during burning, showing expected intrinsic fire safety. Thermal, mechanical properties, and flame retardancy of the reprocessed EVs after hot pressing were very close to those of the original EVs, which was attributed to the sufficient reversibility of APNs. Accordingly, combining the aforementioned features, EVs were utilized to construct alertable-yet-extinguishable fire alarms triggered by either direct flame conduct or high temperature. This work proposed a facile and feasible strategy for designing vitrimers with promising comprehensive properties, which possessed the further potential for other stimuli-responsive materials through phosphate transesterification.

## 4. Materials and Methods

### 4.1. Synthesis of [Bmim]DPPOO

**Scheme 1:**
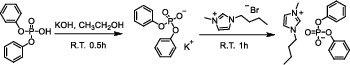
The synthesis route of [Bmim]DPPOO.

The ionic liquid [Bmim]DPPOO was synthesized by an ion-exchange reaction between the commercial ionic liquid 1-butyl-3-methylimidazolium bromide (21.9 g and 0.1 mol) and diphenyl hydrogen phosphate (25.0 g and 0.1 mol), as illustrated in Scheme 1. FT-IR and NMR measurements were carried out to confirm the chemical structures of [Bmim]DPPOO. The FT-IR spectrum of [Bmim]DPPOO was shown in Figure S1(b). Hydroxyl stretching vibration peaks appeared at approximately 3300 cm^−1^. The absorption peaks at 3069 cm^−1^ corresponded to the C-H stretching vibration of benzene rings, while the peaks at 1590 cm^−1^ and 1498 cm^−1^ indicated the existence of the benzene skeleton. Furthermore, the peaks at 1257 cm^−1^ and 1024 cm^−1^ were ascribed to the vibrations of P=O and P-O-C bonds, respectively. The ^1^H NMR spectra of [Bmim]DPPOO (Figure S1(c)) indicated that the chemical shifts at 9.21 ppm and 7.75 ppm were assigned to the H atoms of the imidazole ring; 7.40-6.75 ppm presented the H atom on the benzene rings; 4.15, 1.88, 1.25, and 0.89 ppm referred to the H atoms of the butyl substituent, and the peak of 3.84 ppm corresponded to the methyl substituent of the imidazole ring. In Figure S1(d), the chemical environment of P was determined by ^31^P NMR, which illustrated a chemical shift of -11.54 ppm, verifying a typical phosphate structure.

### 4.2. Preparation of EVs

The curing process of the EVs was initiated from the anionic ring-opening reaction. Specifically, as a curing agent, [Bmim]DPPOO was mixed with DGEBA in a 100 ml round-bottomed flask at proportions of 5 wt%, 10 wt%, and 15 wt%. Accordingly, EVs were named 5% vitrimer, 10% vitrimer, and 15% vitrimer for further research. After removing the bubbles in a vacuum oven at 60°C for 15 min, the liquid mixtures were poured into preheated Teflon molds for procuring (160°C/2 h). Finally, the film specimens were fabricated utilizing stainless steel molds (with thinness of 0.3 mm) on a plate vulcanizer at 160°C/4 h and 180°C/2 h with 3 MPa. For comparison, reference EP thermosets cured with DDM were used as reference samples. A stoichiometric amount of DDM and DGEBA were mixed in a 250 ml round-bottomed flask equipped with a magnetic stirrer at 100°C until homogeneously dispersed. Then, the prepolymers were degassed in a vacuum oven at 60°C for 15 min and poured into preheated Teflon molds. Afterward, the curing process was conducted on a plate vulcanizer at 100°C/1 h, 160°C/2 h, and 180°C/2 h with 3 MPa.

### 4.3. Fabrication of the Fire Alarm Device

An electrical circuit including a DC power supply (4 × 1.5 V), 100 *Ω* resistor, luminous diode, cables, and 15% vitrimer specimens was designed as fire alarm devices. The specimens were warped with aluminum foil for electrical conductivity. The flame-triggered experiments were conducted with a butane blowtorch apparatus (the temperature of the external flame is 800~1300°C).

## Data Availability

All data needed to evaluate the conclusions in the paper are present in the paper and/or the supplementary materials.

## Supplementary Materials

Supplementary Materials Figure S1: the characteristic of [Bmim]DPPOO: digital photograph at room temperature (a). ^1^H NMR spectrum (b). FT-IR spectrum (c). ^31^P NMR spectra (d). Figure S2: curing behaviors: nonisothermal DSC curves at various heating rates (5, 10, 15, and 20°C·min^−1^) for 5% vitrimer, 10% vitrimer, and 15% vitrimer (a). Plots of ln (*β*/T_p_^2^) and ln*β* against 1/T_p_ (b). Evolution of the rheological parameters during the isothermal curing process at 180°C for 5% vitrimer, 10% vitrimer, and 15% vitrimer (c). Figure S3: high-resolution XPS spectra for 15% vitrimer of N 1 s (a) and P 2p (b). Figure S4: digital images of EVs cured with various ratios of [Bmim]DPPOO: pale yellow for 5% vitrimer, orange for 10% vitrimer, and dark brown for 15% vitrimer (a). Swelling experiments using typical solvents on 15% vitrimer (b). Figure S5: stress relaxation curves at various temperatures for 15% vitrimer (a). Linear regression results of natural logarithm relaxation time ln(*τ*) versus reciprocal of temperature 1000/*T* Arrhenius plots (b). Figure S6: the multiple reprocessing of 15% vitrimer from the third time to the sixth time. Figure S7: the reprocessed 5% vitrimer from the ground powder. Figure S8: TGA results of the original and reprocessed 10% vitrimer under N_2_ atmosphere. Figure S9: *T*_g_*s* of EVs cured with different proportions of [Bmim]DPPOO during in DSC heating scan. Figure S10: the MS analysis of the ring-opening product *β*-hydroxy phosphate and the transesterification products. Figure S11: the stress-strain curves of the original and the self-healed EVs. Scheme S1: curing process and primary structures of the cured EVs. Table S1: swelling ratio of 15% vitrimer in various solvents. Table S2: the summarized thermogravimetric data of the EVs and DDM/EP. Table S3: thermal-mechanical behaviors of the EVs in DMA tests. Table S4: cone calorimetric results of DDM/EP, 10% vitrimer, and 15% vitrimer. Table S5: the corresponding parameters in calculating *T*_*v*_. Table S6: the summarized data for the original and reprocessed EVs in tensile tests. Video S1: fire alarm process (2x).mp4.
