# Supplementary material for: Adaptable Phosphate Networks towards Robust, Reprocessable, Weldable, and Alertable-Yet-Extinguishable Epoxy Vitrimer
Source: Research (Wash D C). 2022 Oct 6;2022:9846940. doi: 10.34133/2022/9846940 (PMC9575472; doi:10.34133/2022/9846940)
Supplement: Supplementary Materials — Figure S1: the characteristic of [Bmim]DPPOO: digital photograph at room temperature (a). 1H NMR spectrum (b). FT-IR spectrum (c). 31P NMR spectra (d). Figure S2: curing behaviors: nonisothermal DSC curves at various heating rates (5, 10, 15, and 20°C·min−1) for 5% vitrimer, 10% vitrimer, and 15% vitrimer (a). Plots of ln (β/Tp2) and lnβ against 1/Tp (b). Evolution of the rheological parameters during the isothermal curing process at 180°C for 5% vitrimer, 10% vitrimer, and 15% vitrimer (c). Figure S3: high-resolution XPS spectra for 15% vitrimer of N 1 s (a) and P 2p (b). Figure S4: digital images of EVs cured with various ratios of [Bmim]DPPOO: pale yellow for 5% vitrimer, orange for 10% vitrimer, and dark brown for 15% vitrimer (a). Swelling experiments using typical solvents on 15% vitrimer (b). Figure S5: stress relaxation curves at various temperatures for 15% vitrimer (a). Linear regression results of natural logarithm relaxation time ln(τ) versus reciprocal of temperature 1000/T Arrhenius plots (b). Figure S6: the multiple reprocessing of 15% vitrimer from the third time to the sixth time. Figure S7: the reprocessed 5% vitrimer from the ground powder. Figure S8: TGA results of the original and reprocessed 10% vitrimer under N2 atmosphere. Figure S9: Tgs of EVs cured with different proportions of [Bmim]DPPOO during in DSC heating scan. Figure S10: the MS analysis of the ring-opening product β-hydroxy phosphate and the transesterification products. Figure S11: the stress-strain curves of the original and the self-healed EVs. Scheme S1: curing process and primary structures of the cured EVs. Table S1: swelling ratio of 15% vitrimer in various solvents. Table S2: the summarized thermogravimetric data of the EVs and DDM/EP. Table S3: thermal-mechanical behaviors of the EVs in DMA tests. Table S4: cone calorimetric results of DDM/EP, 10% vitrimer, and 15% vitrimer. Table S5: the corresponding parameters in calculating Tv. Table S6: the summarized data for the orig [file 9846940.f1.zip › RESEARCH-D-22-00739 - Supplementary Materials.docx]

Adaptable Phosphate Networks towards Robust, Reprocessable, Weldable, and Alertable-yet-extinguishable Epoxy Vitrimer

*Jia-Hui Lu, Zhen Li, Jia-Hui Chen, Shu-Liang Li, Jie-Hao He,Song Gu, Bo-Wen Liu, Li Chen*, Yu-Zhong Wang**

School of Chemical Engineering, The Collaborative Innovation Center for Eco-Friendly and Fire-Safety Polymeric Materials (MoE), National Engineering Laboratory of Eco-Friendly Polymeric Materials (Sichuan), State Key Laboratory of Polymer Materials Engineering, Sichuan University, Chengdu 610064, China

E-mail: l.chen.scu@gmail.com (LC); yzwang@scu.edu.cn (YZW).

**Supplementary Materials**

**Materials**

Diphenyl hydrogen phosphate (98%), 1-butyl-3-methylimidazolium bromide (99%), and 4,4′-diaminodiphenyl methane (DDM, 99%) were purchased from Adamas (Shanghai China); epichlorohydrin (ECH, 99%) was provided by Aladdin Chemical Reagent Co., Ltd. (Shanghai, China); potassium hydroxide (AR) was procured from Fuchen Chemical Reagent Co., Ltd. (Tianjin, China); ethanol (99.7%) was supplied by Haixing Chemical Reagent Factory (Sichuan, China); and diglycidyl ether of bisphenol A (DGEBA, E51) with an epoxide equivalent weight of 196 g·mol^-1^ was supplied by Nantong Xingchen Synthetic Material Co., Ltd. (Nantong, China). All the chemicals were utilized as received.

**Characterization**

^1^H and ^31^P NMR spectra were obtained with a Bruker AVANCE Ⅲ HD–400 NMR instrument (Bruker, Germany), where *d_6_*-dimethyl sulfoxide (DMSO-*d_6_*) was used as the solvent.

Fourier transform infrared (FT-IR) spectra were recorded between 500 cm^-1^ and 4000 cm^-1^ (using KBr pellets) on an infrared spectrophotometer (Nicolet 6700, Thermo Fisher Scientific, USA).

Curing behavior was monitored by differential scanning calorimetry (DSC Q 200, TA, USA) with the non-isothermal heating procedure at 5, 10, 15, and 20 ºC⋅min^−1^. The glass-transition temperature of the EVs was tested through DSC method with a heating rate of 10 ºC⋅min^−1^.

The gelation process was investigated by a dynamic rotational rheometer (ARES Rheometer, TA, USA) with disposable aluminum parallel plates (20 mm in diameter with a gap of 0.1 mm). The experiments were performed at a frequency of 1 Hz and an amplitude of 10%.

The swelling tests of 15% Vitrimer were conducted by utilizing different solvents, including tetrahydrofuran, acetone, chloroform, *N, N*-dimethylformamide, deionized water, and dimethyl sulfoxide. The swelling test was carried out following the steps below: The cured EVs were cut into pieces, swelled by the above-mentioned solvent, and extracted for 24 h under room temperature.

Gel content was evaluated with a Soxhlet extractor at 60 °C for 48 h by using acetone as a solvent. After swelling or gel content experiments, the sample was dried in a vacuum oven at 50 °C for 24 h until constant weight. The mass of the original samples (m_0_), the swelled samples (m_1_), and the dried samples (m_2_) were recorded for calculating the swelling ratio (S (%)), and gel content (G (%)) which were determined by Equations S(1) and S(2):

$$\begin{aligned} \text{S (\%)=}\frac{m_{1}}{m_{0}}\times100\%\#S\text{(1)} \end{aligned}$$

$$\begin{aligned} \text{G (\%)=}\frac{m_{2}}{m_{0}}\times100\%\#S\text{(2)} \end{aligned}$$

X-ray photoelectron spectroscopy (XPS) of the EVs was performed with XSAM 800 spectrometer (KRATOS, UK) using Al Ka excitation radiation (1486.6 eV).

Thermal stability was estimated via thermogravimetry analysis (TGA 5500, TA Instruments, USA) under N_2_ or air atmospheres from 40 °C to 700 °C with a heating rate of 10 °C·min^-1^.

Thermomechanical behaviors under tensile mode were performed on a dynamic mechanical analysis machine (DMA, Q 800, TA Instruments, USA) with a heating rate of 5 °C·min^-1^ at a constant frequency of 1.0 Hz and oscillation amplitude of 10.0 μm. The crosslinking density of the epoxy vitrimer was determined according to the rubbery plateau modulus in the DMA tests and was calculated from Equation S(3):

$$\begin{aligned} \text{υ}\text{e}\text{=}\frac{\text{E}\text{r}}{\text{3RT}}\#S\text{(3)} \end{aligned}$$

where *E*_r_ refers to the storage modulus of the rubbery plateau (at a temperature of *T*_α_ + 30 °C), *R* represents the gas constant (8.314 J·K^-1^·mol^-1^), and *T* is the absolute temperature.

Tensile tests of the EVs were conducted with an electronic universal material testing machine (INSTRON 3366, INSTRON, USA) at a strain rate of 2 mm·min^-1^ at room temperature. Each sample was tested at least five times, and the results reported were average values of each group with error bars.

FTLR T420 (FTLR) was used to evaluate the temperature evolution of 15% Vitrimer heated by an alcohol lamp.

Flame retardancy was evaluated by limiting the oxygen index (LOI) on a JF-3 oxygen index meter (Jiangning, China) according to ASTM D2863-19, with a bar of 130 mm × 6.5 mm × 3.2 mm. Burning behaviors were recorded by a cone calorimeter (iCone, Fire Testing Technology, UK) according to ISO 5660-1. The specimens with a size of 100 mm × 100 mm × 3 mm were exposed to a radiant cone in a heat flux of 35 kW·m^-2^. Raman spectroscopy of the burning residual after cone calorimetry was performed on a Raman imaging microscope (DXR2xi, Thermo Fisher Scientific, USA) with a 532 nm laser.

The shape memory behaviors were measured by DMA equipment using a force-controlled mode. The detailed procedures and evaluation formulas are also listed behind. The self-healing behavior was recorded on a polarizing microscope with a heating stage (Axio Scope. A1, Carl Zeiss, Germany).

Stress relaxation tests were conducted on the DMA machine using tensile mode. Specimens with dimensions of 25 mm × 4~6 mm × 0.20~0.25 mm were stretched to a constant strain of 2% during the measurement. The force was applied for 5 min followed by a 60 min recovery period.

The welding performance was performed with two overlaid rectangular film specimens sandwiched by pieces of sheet metal treated with a conventional oven at 190 °C for 0.5 h.

For the reprocessing procedure, the samples were cut into small pieces or ground into powder before being hot pressed using a plate vulcanizer at 190 °C/0.5 h with a pressure of 3 MPa. After cooling to room temperature, the recycled specimens were obtained.

A digital photograph of [Bmim]DPPOO in a viscous liquid state at room temperature was pictured in Figure S1(a); FT-IR spectrum, ^1^H, and ^31^P NMR spectra of [Bmim]DPPOO were shown in Figure S1(b)-(d), suggesting the successful synthesis of [Bmim]DPPOO.

Figure S1: The characteristic of [Bmim]DPPOO: Digital photograph at room temperature (a). ^1^H NMR spectrum (b). FT-IR spectrum (c). ^31^P NMR spectra (d).

**Curing behavior of the EVs**

Curing behavior was monitored by differential scanning calorimetry (DSC) with the non-isothermal heating procedure at 5, 10, 15, and 20 ºC⋅min^−1^, and the curing activation energy was calculated based on the Kissinger equation (Equation S(4)) and Flynn-Wall-Ozawa equation (Equation S(5)).

Kissinger equation:

$$\begin{aligned} \text{ln}\frac{\text{β}}{\text{T}_{\text{p}}^{\text{2}}}\text{=}\text{ln}\frac{\text{A·R}}{\text{E}_{\text{a}}}\text{-}\frac{\text{E}_{\text{a}}}{\text{R}}\text{×}\frac{\text{1}}{\text{T}_{\text{p}}}\#\#\#S\text{(4)} \end{aligned}$$

Flynn-Wall-Ozawa equation:

$$\begin{aligned} \text{l}\text{nβ+1.0516×}\frac{\text{E}_{\text{a}}}{\text{nR}}\text{=C’}\#S\#\#\text{(5)} \end{aligned}$$

The reaction activation energy (*E*_a_) was in kJ·mol^-1^, β represents the heating rate in K·min^-1^; *T*_p_ is the peak temperature of the exothermic curves in K; C, and C’ were constants.

Figure S2: Curing behaviors: DSC curves at various heating rates (5, 10, 15, 20 ºC·min^-1^) for 5% Vitritimer, 10% Vitritimer, and 15% Vitritimer (a). Plots of ln (β/T_p_^2^) and lnβ against 1/T_p_ (b). Evolution of the rheological parameters during the isothermal curing process at 180 ºC for 5% Vitritimer, 10% Vitritimer, and 15% Vitritimer (c).

Figure S3: High-resolution XPS spectra for 15% Vitrimer of N 1s (a) and P 2p (b).

Scheme S1: Curing process and primary structures of the cured EVs.

Figure S4: Digital images of EVs cured with various ratios of [Bmim]DPPOO: pale yellow for 5% Vitrimer, orange for 10% Vitrimer, and dark brown for 15% Vitrimer (a). Swelling experiments using typical solvents on 15% Vitrimer (b). Gel contents of the EVs (c).

**Raman spectroscopy analysis**

Raman spectra of the burning residues showed two broad and strongly overlapping bands with an intensity maximum at around 1350 cm^-1^ (D band) and 1580 cm^-1^ (G band), as shown in Figure 2(f) in the main text. Accordingly, the D band reflects the free bond vibration of the disordered graphite or amorphous carbon structures, which was considered defects of graphite materials. Whereas the G band contributes to an E_2g_ mode of the hexagonal graphite and is related to the vibration of the sp^2^ orbital. Therefore, the relative intensity of the D band and the G band (I_D_/I_G_) correlates to the size of carbonaceous microstructures of the as-formed residues [1, 2].

**Stress relaxation**

The relaxation time (*τ^*^*) is defined as the time when *G(t)/G*_0_ equals 1/*e* [3]*.* In this work, the value of *τ^*^* significantly reduced at elevated temperature because the phosphoester-exchange rate was accelerated, as shown in Figure S5(a). The activation energy (*E*_a_) reflects the capability of thermoreversible rearrangement of the dynamic covalent networks and can be calculated by the Arrhenius equation (Equation S(6)).

$$\begin{aligned} \text{τ}^{\text{*}}\text{=}\text{τ}_{\text{0}}\exp\left( \frac{\text{E}_{\text{a}}}{\text{RT}} \right)\#S\text{(6)} \end{aligned}$$

Where *τ*_0_ is the relaxation time at infinite temperature, *T* is the experimental temperature, *E*_a_ is the activation energy of the reversible bond exchange process, while *R* refers to the universal gas constant. Our calculated data from Figure S5(b) showed that the activation energy of phosphoester exchange reaction of 15% Vitrimer was 86.4 kJ·mol^-1^, by fitting the linear regression results of *ln(τ^*^)* verse 1000/*T*.

Figure S5: stress relaxation curves at various temperatures for 15% Vitrimer (a). Linear regression results of natural logarithm relaxation time ln(*τ*) versus reciprocal of temperature 1000/*T* Arrhenius plots (b).

*T*_v_, defined as the topology freezing transition temperature, acts as the critical point for the viscoelastic response of vitrimer [4]. The value of *T*_v_ is usually considered as the temperature at which the viscosity is beyond 10^12^ Pa·s. We calculated the *T*_v_ according to the Arrhenius equation and Maxwell equation in Equations S(7) - S(10). Here, *T*_v_ was calculated to be 91.4°C, which was slightly higher than *T*_g_ of 15% vitrimer. Thus, the epoxy vitrimer behaved like typical thermosets below *T*_g_ and demonstrated preferable stimuli-response behavior, malleability, and reprocessibility above *T*_v_.

$$\begin{aligned} \text{k=A}\text{e}^{\frac{\text{-}\text{E}\text{a}}{\text{RT}}}\#\text{S(7)} \end{aligned}$$

$$\begin{aligned} \ln\left( \text{τ}^{\text{*}} \right)\text{=}\frac{\text{E}_{\text{a}}}{\text{RT}}\text{+}\ln\left( \text{τ}_{\text{0}} \right)\#\text{S(8)} \end{aligned}$$

$$\begin{aligned} \text{η=}\frac{\text{τ}\text{E}^{\text{'}}}{\text{2}\left( \text{1+v} \right)}\#\text{S(9)} \end{aligned}$$

$$\begin{aligned} \text{y=10.39x-18.87}\#\text{S(10)} \end{aligned}$$

**Reprocessing**

The multiple reprocessing cycles of 15% Vitrimer hot-pressed from the third time to the sixth time were pictured in Figure S6. The reprocessed 5% Vitrimer from the ground powder was also illustrated in Figure S7. Afterward, tensile properties, including tensile strength and modulus, and elongation at break of the original and reprocessed 15% Vitrimer and 10% Vitrimer were summarized in Table S6.

Figure S6: The multiple reprocessing of 15% Vitrimer from the third time to the sixth time.

Figure S7: The reprocessed 5% Vitrimer from the ground powder.

Figure S8: TGA results of the original and reprocessed 10% Vitrimer under N_2_ atmosphere.

**Welding and shape memory property**

Figure S9: *T*_g_s of EVs cured with different proportions of [Bmim]DPPOO during in DSC heating scan.

The shape memory property of EP vitrimer was evaluated by tensile tests with DMA-controlled force mode. The programmed step was set as follows: After equilibrium at a Thigh (90~120 °C) for 10 min, the strain was recognized as ε_begin_.

Then, the rectangular specimen was stretched to a set force of 0.5 MPa at 0.1 MPa·min^-1^, and the strain was *ε*_deform_.

Afterward, the sample was cooled to *T*_low_ (30 °C) under a constant load to fix the temporary shape for 10 min. After removing the load at *T*_low_, the strain was recorded as *ε*_fix_.

Ultimately, the permanent shape was recovered by reheating to the *T*_high_ and isothermally treated for 10 min, where the strain was recorded as *ε*_final_.

The subsequent shape memory cycles followed the same parameters described above.

Here, the fixing ratio (*R*_f_) and the recovery ratio (*R*_r_) are two important parameters to assess shape memory behaviors. Further, the corresponding calculated formulas are listed as follows:

$$\begin{aligned} \text{R}\text{f}\text{=}\frac{\text{ε}_{\text{fix}}}{\text{ε}_{\text{deform}}}\text{×100\%}\#\text{S(11)} \end{aligned}$$

$$\begin{aligned} \text{R}\text{r}\text{=}\frac{\text{ε}_{\text{deform}}\text{-}\text{ε}_{\text{final}}}{\text{ε}_{\text{deform}}\text{-}\text{ε}_{\text{begin}}}\text{×100\%}\#\text{S(12)} \end{aligned}$$

**HPLC analysis**

To confirm the chemical structures of the model reactions and further investigate the phosphate transesterification mechanism, LC-MS (ESI source) was also conducted using an Agilent LC1290-QQQ-6470 instrument in positive mode. As illustrated in Figure S10, the chemical structures of the diphenyl hydrogen phosphate and the ring-opening product β-hydroxy phosphate were recorded. After thermal treatment at 90 ºC for 4 hours, transesterification reactions occurred based on the internal participated mechanism where new phosphate structure 2 (m/z=267) and structure 3 (m/z=215) were detected, which confirmed the inferences in the NMR measurement.

Figure S10: The MS analysis of the ring-opening product β-hydroxy phosphate and the transesterification products.

**Self-healing**

To investigate the self-healing efficiency, each epoxy vitrimer specimen was cut into two fragments, overlapped to contact with each other, sandwiched between two stainless steel plates, and thermally treated at 190 °C for 0.5 h. After that, the healed epoxy vitrimers were subjected to tensile experiments; and the stress-strain curves before and after self-healing were illustrated in Figure S11 in the Supplementary Materials. Tensile strength and the elongation at break of the healed 5% Vitrimer were 41.4 MPa and 2.1%, respectively, which showed poor self-healing efficiency with a recovery ratio of 66.7%, compared to the original ones. With increasing contents of [Bmim]DPPOO, self-healing performance was remarkably enhanced. The stress-strain curves of 10% Vitrimer after self-healing almost overlapped with that of the original samples and the self-healing efficiency was calculated to be 96.7%. Self-healed 15% Vitrimer demonstrated increased tensile strain and slightly declined strength since the excellent weldability resulted in the dimensional change on the overlapped areas, which were expected to withstand larger tensile loads than the original samples. Moreover, the tensile modulus of 15% Vitrimer was comparable to the original ones, indicating the mechanical property of 15% Vitrimer after self-healing was maintained.

Figure S11: The stress-strain curves of the original and the self-healed EVs.

**Supplementary Tables**

Table S1. Swelling ratio of 15% Vitrimer in various solvents.

| Solvent | Swelling ratio (%) |
| --- | --- |
| tetrahydrofuran | 122.2 ± 3.1 |
| acetone | 129.5 ± 1.6 |
| chloroform | 122.5 ± 1.1 |
| *N,N*-dimethylformamide | 144.5 ± 3.8 |
| dimethyl sulfoxide | 151.7 ± 2.6 |
| deionized water | 104.4 ± 2.2 |

Table S2. The summarized thermogravimetric data of the EVs and DDM/EP.

| Sample | N_2_ | | | Air | | |
| --- | --- | --- | --- | --- | --- | --- |
|  | T_5%_^a)^  (°C) | T_max_^b)^  (°C) | Residues^c)^  (wt%) | T_5%_  (°C) | T_max_  (°C) | Residues  (wt%) |
| 5%  Vitrimer | 357.2 | 399.2 | 21.3 | 341.6 | 392.0 | 2.3 |
| 10% Vitrimer | 339.0 | 383.9 | 21.6 | 339.2 | 382.7 | 4.8 |
| 15% Vitrimer | 328.2 | 371.9 | 22.2 | 328.6 | 373.7 | 15.5 |
| DDM/EP | 371.2 | 389.7 | 17.1 | 384.4 | 393.6 | 0.7 |

^a)^ The temperature at 5% weight loss;

^b)^ The temperature at maximum decomposition rate;

^c)^ The residual weight at 700 °C.

Table S3. Thermal-mechanical behaviors of the EVs in DMA tests.

|  | E’ at glassy state  (MPa) | *T*_α_  (°C) | E’ at Rubbery plateau (MPa) | 𝜐_e_  (mol∙m^-3^) |
| --- | --- | --- | --- | --- |
| 5% Vitrimer | 2528.3 | 118.1 | 5.26 | 513.2 |
| 10% Vitrimer | 1646.1 | 109.8 | 2.16 | 217.3 |
| 15% Vitrimer | 1226.7 | 101.2 | 1.36 | 138.5 |

Table S4. Cone calorimetric results of DDM/EP, 10% Vitrimer, and 15% Vitrimer.

| Samples | TTI  (s) | pHRR  (kW·m^-2^) | THR  (MJ·m^-2^) | pSPR  (m^2^·s^-1^) | pCO  (g·s^-1^) | MAHRE  (kW·m^-2^) |
| --- | --- | --- | --- | --- | --- | --- |
| DDM/EP | 73 | 1413 | 91.6 | 0.96 | 0.22 | 353.0 |
| 10% Vitrimer | 43 | 515 | 71.0 | 0.75 | 0.18 | 272.8 |
| 15% Vitrimer | 37 | 357 | 57.6 | 0.55 | 0.12 | 237.8 |

Table S5. The corresponding parameters in calculating *T*_v_.

| Temperature (°C) | Temperature (K) | 1000/T (K) | τ^*^ (s) | lnτ^*^ |
| --- | --- | --- | --- | --- |
| 180 | 453.15 | 2.21 | 3523 | 4.07 |
| 190 | 463.15 | 2.16 | 1962 | 3.49 |
| 200 | 473.15 | 2.11 | 1426 | 3.17 |
| 210 | 483.15 | 2.07 | 713 | 2.48 |
| 220 | 493.15 | 2.03 | 499 | 2.12 |

Table S6. The summarized data for the original and reprocessed EVs in tensile tests.

| Samples | | Tensile strength (MPa) | Elongation at break (%) | Tensile modulus (MPa) |
| --- | --- | --- | --- | --- |
| 10% Vitirmer | original | 74.0±5.5 | 7.2±1.2 | 1916.9±351.8 |
|  | reprocessed | 87.8±12.3 | 5.5±0.4 | 2975.3±363.6 |
| 15% Vitirmer | original | 64.7±6.4 | 10.1±1.5 | 1360.1±215.5 |
|  | reprocessed | 62.5±5.5 | 9.6±0.9 | 1433.6±138.2 |

**Flame-triggered alarm**

The fire alarm process of 15% Vitrimer initiated by butane blowlamp flame was shown in Supplementary Video named “Video - S1- Fire Alarm Process (2X).mp4”.

**References**

[1] A. Sadezky, H. Muckenhuber, H. Grothe, R. Niessner, U. Pöschl, "Raman microspectroscopy of soot and related carbonaceous materials: Spectral analysis and structural information," *Carbon*, vol. 43, no. 8, pp. 1731-1742, 2005. <http://doi.org/10.1016/j.carbon.2005.02.018>

[2] J. Schwan, S. Ulrich, V. Batori, H. Ehrhardt, S.R.P. Silva, "Raman spectroscopy on amorphous carbon films," *Journal of Applied Physics*, vol. 80, no. 1, pp. 440-447, 1996. <http://doi.org/10.1063/1.362745>

[3] L. Zhang, S.J. Rowan, "Effect of sterics and degree of cross-linking on the mechanical properties of dynamic poly(alkylurea–urethane) networks," *Macromolecules*, vol. 50, no. 13, pp. 5051-5060, 2017. <http://doi.org/10.1021/acs.macromol.7b01016>

[4] H. Fang, W. Ye, Y. Ding, H.H. Winter, "Rheology of the critical transition state of an epoxy vitrimer," *Macromolecules*, vol. 53, no. 12, pp. 4855-4862, 2020. <http://doi.org/10.1021/acs.macromol.0c00843>
